# Comparison of Fatty Acid Proportions Determined by Mid-Infrared Spectroscopy and Gas Chromatography in Bulk and Individual Milk Samples

**DOI:** 10.3390/ani10061095

**Published:** 2020-06-25

**Authors:** Eva Samková, Jiří Špička, Oto Hanuš, Petr Roubal, Lenka Pecová, Lucie Hasoňová, Pavel Smetana, Marcela Klimešová, Jindřich Čítek

**Affiliations:** 1 Department of Food Biotechnologies and Agricultural Products Quality, Faculty of Agriculture, University of South Bohemia in České Budějovice, Studentská 1668, 370 05 České Budějovice, Czech Republic; pecovl00@zf.jcu.cz (L.P.); hasonova@zf.jcu.cz (L.H.); smetana@zf.jcu.cz (P.S.); 2 Department of Applied Chemistry, Faculty of Agriculture, University of South Bohemia in České Budějovice, Studentská 1668, 370 05 České Budějovice, Czech Republic; spicka@zf.jcu.cz; 3 Dairy Research Institute, s.r.o., Ke Dvoru 12a, 160 00 Prague 6, Czech Republic; hanus.oto@seznam.cz (O.H.); roubal@milcom-as.cz (P.R.); marcela.vyletelova@seznam.cz (M.K.); 4 Department of Genetics and Plant Production, Faculty of Agriculture, University of South Bohemia in České Budějovice, Studentská 1668, 370 05 České Budějovice, Czech Republic; citek@zf.jcu.cz

**Keywords:** dairy cow, Czech Fleckvieh, Holstein, raw milk, fatty acids, mid-infrared spectroscopy, gas chromatography, regression analysis, correlation coefficient

## Abstract

**Simple Summary:**

Information about fatty acid proportions in milk fat is important for many purposes, such as animal breeding, animal health control, as well as human nutrition. The routine methods for determining fatty acid proportions (e.g., mid-infrared spectroscopy) are rapid and relatively cheap, but there is a need to compare them with the reference analytical method (gas chromatography) to ensure their validity and suitability for various milk samples. The aim of this study is to compare the proportions of single fatty acids and their sums determined by utilizing both of these analytical methods and the resulting correlation coefficients. Our results show that the mid-infrared spectroscopy method is more appropriate (both for bulk and individual milk samples) for fatty acids present in high proportions of the total fat and for the sum of fatty acids (such as saturated and unsaturated) than for fatty acids with low proportions.

**Abstract:**

Rapid analytical methods can contribute to the expansion of milk fatty acid determination for various important practical purposes. The reliability of data resulting from these routine methods plays a crucial role. Bulk and individual milk samples (60 and 345, respectively) were obtained from Czech Fleckvieh and Holstein dairy cows in the Czech Republic. The correlation between milk fatty acid (FA) proportions determined by the routine method (infrared spectroscopy in the mid-region in connection with Fourier transformation; FT-MIR) and the reference method (gas chromatography; GC) was evaluated. To validate the calibration of the FT-MIR method, a linear regression model was used. For bulk milk samples, the correlation coefficients between these methods were higher for the saturated (SFAs) and unsaturated FAs (UFAs) (r = 0.7169 and 0.9232; *p* < 0.001) than for the *trans* isomers of UFAs (TFAs) and polyunsaturated FAs (PUFAs) (r = 0.5706 and 0.6278; *p* < 0.001). Similar results were found for individual milk samples: r = 0.8592 and 0.8666 (*p* < 0.001) for SFAs and UFAs, 0.1690 (*p* < 0.01) for TFAs, and 0.3314 (*p* < 0.001) for PUFAs. The correlation coefficients for TFAs and PUFAs were statistically significant but too low for practical analytical application. The results indicate that the FT-MIR method can be used for routine determination mainly for SFAs and UFAs.

## 1. Introduction

Comprehensive knowledge of the composition and properties of milk provides useful insight for many purposes, such as ensuring milk quality, animal health management, dairy cattle breeding, and benefiting the dairy industry in general. Ensuring high-quality milk is also important for consumers who, nowadays, are more interested in their health. Due to the rapid development of software and hardware for infrared (IR) spectroscopy (MIR, FT-MIR, FT-NIR), as well as other routine methods (e.g., ultrasound analysis), there has been an increase in the availability and use of analytical methods in the dairy industry [1]. MIR represents IR spectroscopy in the mid-region, with optical filter technology. FT-MIR represents IR spectroscopy of the whole spectrum in the mid-region through the Michelson interferometer and Fourier transformations, while FT-NIR is similar to FT-MIR represented in the near IR range (see, e.g., [2,3,4,5,6,7,8,9]).

These analyzers are used in dairy laboratories with different types of modifications according to the analytical techniques of the manufacturers (Foss Electric from Denmark, Bentley Instruments from the USA, Delta Instruments from the Netherlands, and others). Recently, in addition to the basic components [2,7] of milk (fat, protein, casein, lactose, total solids, solids-non-fat), the analysis spectrum has been expanding, primarily with undesirable metabolites (citric acid, free fatty acids (FAs)), ketones (acetone, beta-hydroxybutyric acid), urea [5,10,11,12,13,14], and other components in milk, especially the milk FA proportion (MFAP) [3,15,16,17]. The monitoring of the above-listed milk components and metabolites serve to control the health, breeding, nutrition, and reproduction of cows as well as the quality of their milk [5,12,16,18,19,20,21,22].

The composition of milk fat was previously monitored mainly due to the assessment of the nutritional properties of milk and its potential health benefits/risks for consumers and its technological properties, which affect milk processing [1,23,24,25]. Recently, the FA proportion in the milk fat has also been considered in connection with the metabolic status of dairy cows, the prediction of milk quality from different farm production systems, geographical origins, and the contribution of dairy cows to climate change (methane emissions) [26,27,28,29].

The reliability of results when determining the milk fat composition through routine methods is important for the practical and effective use of these methods. In general, the quality level of the calibrations for routine (indirect) methods (IMs), according to the results of reference (direct) methods (DMs), is essential for the reliability of the obtained analytical results. In practice, MIR and NIR are most frequently used as IM, while gas chromatography (GC) is usually used as DM, and the comparison of results between both methods is still current. The procedures are still evaluated according to the various analyzed materials (types of milk or dairy products), calibrations, and conditions of their own measurements (see, e.g., [4,6,7,30,31]).

Based on the assessment of the results of these calibrations, in addition to the MFAP results in the field cow trial, Soyeurt et al. [32] recommended that the obtained results are a possible basis for the breeding and feeding of dairy cows for improved milk fat (i.e., higher proportion of monounsaturated FAs and lower proportion of saturated FAs). Similarly, with the FA proportions, IM (FT-MIR) calibrations were developed and carried out according to the DM results for different milks (cow, sheep, goat) and various dairy products, as well as their various components and properties, and these have been continuously validated by some authors [6,33]. Further, using FT-NIR, the major constituents of milk (such as fat, proteins (true and crude), casein, lactose, total solids, solids-non-fat), dairy foods (cheeses—free amino acids and their ripening process, yogurt, and cream), and the falsification of milk by foreign milk addition were evaluated [4,26,30,31,34,35].

Coppa et al. [6] have compared the result reliability for MIR and NIR MFAP determination. The NIR showed worse predictions than MIR for almost all FAs when expressed as g/kg of milk. The NIR predictions on fresh liquid and oven-dried milk were similar, but the reliability decreased for thawed liquid milk. The high performance shown by NIR and MIR allows for their use in routine MFAP recordings. Nevertheless, this IM (FT-NIR) usually gives results with a slightly less tight relationship to DM (in terms of analysis of liquid, raw, and consumer milk) than for the MIR- and FT-MIR-specialized methods with flow measurement cells for milk due primarily to the absence of the mechanical homogenization procedure for milk fat globules in most of the FT-NIR instruments.

However, these results are still very good for practical applications. Among other things, the FT-NIR technique is also used in the so-called real-time analysis [36,37] for continuous measurement of the components and properties (e.g., fat, proteins, lactose, somatic cell counts) of the milk flowing directly during milking. The research and development of these calibration procedures in dairy IM analysis (especially IR spectroscopy) is methodologically very important for following research and practical official applications of milk analysis results [28,38] in dairy systems, for instance, the genetic improvement of cattle, the health control of dairy cows, and ensuring milk quality [39].

The aim of this work is to evaluate the parameters of FT-MIR calibrations according to the GC results for MFAP measurements and validate the predictive reliability of the routine method under defined experimental conditions to extend the spectrum of relevant analytical and methodological knowledge.

## 2. Materials and Methods

Our research was done within the project No. QJ1510336 of National Agency for Agricultural Research (Národní Agentura pro Zemědělský Výzkum, NAZV) under the Ministry of Agriculture of the Czech Republic, applying methodological demands for animal health protection. Thus, all experiments were performed in accordance with relevant guidelines and regulations recommended by the Ministry of Agriculture of the Czech Republic. In the case of our work, milk samples (both for individual and bulk) were taken from commercial herds and only during the regular testing of milk performance and milk quality, where approval by a properly constituted research ethics committee is not required.

### 2.1. Milk Samples

The milk sample collection was designed to obtain most of the main factors (area, season, breed, parity, and stage of lactation) that affect the MFAPs in both bulk and individual milk samples.

Bulk milk samples (*n* = 60) were collected at 15 dairy cow herds across the Czech Republic, with four samples per year from each herd. Dairy cows of two cattle breeds (Holstein and Czech Fleckvieh) were included in this experiment. The herds included from 35 to 530 dairy cows and were kept at an altitude of 250 to 350 m. The 305-day lactation yields of these herds ranged from 7650 to 11,190 kg of milk. Cow stables utilized free-housing and were equipped with milking parlors (*n* = 14). One herd (35 cows) was equipped with a tie-laying stable and pipeline milking equipment. Dairy cows were fed using a feeding ration widely used in the Czech Republic animal feed sector, and based on preserved forage (silages), hay, and concentrates (grain, minerals, vitamins), according to milk yield (in the form of total mixed ration).

Individual milk samples (*n* = 345) were collected in six herds during the controlled days of regular milk recording. The herds consisted of Holstein dairy cows (*n* = 173) kept in three herds and Czech Fleckvieh dairy cows (*n* = 172) also kept in three herds. The sampling was carried out for different herds during different months of the year.

The obtained milk samples were divided into two portions and transported immediately to the laboratory at 5 °C. The first part of the fresh milk sample was used for the determination of selected milk quality parameters and MFAPs by the IM (FT-MIR). The second part of the sample was used to determine MFAPs by the DM (GC). The samples before GC analysis were preserved by being frozen (−18 °C). No chemical preservatives were used for all samples. The milk samples were gently and thoroughly mixed before each manipulation.

### 2.2. IM (FT-MIR) Analysis

The content of fat, protein, lactose, solids-non-fat, urea, citric acid, beta-hydroxybutyric acid, acetone, and somatic cell counts was determined according to ČSN ISO 8196-1 (570536), ČSN ISO 8196-2 (570536), and ČSN ISO 8196-3 (570536) [40,41,42].

Milk fat, protein, casein, lactose, urea, and MFAP were determined on regularly calibrated and controlled equipment (Milko-Scan FT6000) using MIR prediction models developed and commercialized by FOSS (FOSS Electric A/S, Hillerød, Denmark).

The MFAP was obtained by processing the values according to FOSS Application Note 64 [43]. Thus, 8 groups of FAs (saturated FAs (SFAs), unsaturated FAs (UFAs), monounsaturated FAs (MUFAs), polyunsaturated FAs (PUFAs), *trans* isomers of UFAs (TFAs), short-chain FAs (SCFAs), medium-chain FAs (MCFAs), and long-chain FAs (LCFAs)) and 3 individual FAs (C16:0, C18:0, and C18:1) were obtained.

The unit in the prediction models is g FA per 100 g milk since the Milko-Scan cannot separate the fat from the remaining milk portion. This result can, however, easily be converted to g FA per 100 g total FAs by means of a calculated component, including Milko-Scan fat in the milk prediction model and a conversion factor of 0.95 (from total fat to total FAs). FAs and their groups were calculated as FAs determined by the FT-MIR (g/100 g in milk) × 100/fat determined by the FT-MIR × 0.95 [44].

### 2.3. DM (GC) Analysis

Milk fat was extracted with petroleum ether from freeze-dried milk samples. FAs in extracted fat were re-esterified to their methyl esters with a methanolic solution of potassium hydroxide. Briefly, 0.2 mL 2M KOH in methanol was added to 1 mL petroleum ether extract, and the sample was left for 2 min in a water bath at 60 °C. The sample was then neutralized with 0.4 mL 1M HCl in methanol, diluted with 1 mL petroleum ether, and used for GC analysis. Methyl esters of FAs were determined by GC method (Table 1) using a Varian 3800 apparatus (Varian Techtron, Palo Alto, CA, USA) with FID (for quantitative) and 4000 MS detector (Varian; for qualitative analysis) on a capillary column 50 m × 0.25 mm and 0.25-µm film thickness (SELECT FAME; Varian).

FA proportion is specified by counting peak area proportion to the total peak area of all determined FAs. Weight percentage data were calculated from the area data by means of the relative factors by standards FAME mix (Supelco, Darmstadt, Germany). A total of 56 FAs were determined by the GC method.

### 2.4. Statistical Analysis

Statistica 12.0 (StatSoft 2013) software was used for statistical calculations (descriptive statistics, correlation and regression analysis). Coefficient of variation (relative standard deviation; RSD) was calculated as (standard deviation/mean) × 100. A Student’s *t*-test for the comparison of two means (FT-MIR and GC analysis) was used. Pearson correlation coefficient (r) and Spearman’s rank correlation coefficient (SC) were used at the usual levels of significance (0.05; 0.01; 0.001).

## 3. Results and Discussion

### 3.1. Quality Parameters for Bulk and Individual Milk Samples

The basic statistical characteristics of the milk samples shown in Table 2 indicate a very well-balanced set, with optimal variability of the milk quality parameters. This variability is given by both the range (minimum and maximum values) and the relative standard deviations.

As compared to bulk samples, for all selected milk quality parameters, higher variability was observed for individual samples, as expected. The values found in our study correspond to literature data (e.g., [45,46]) under similar dairy cow rearing conditions. In the monitored set, the RSD for fat content was 11.3% (with a range of minimum and maximum values from 2.57% to 5.15%) for bulk milk samples and 21.2% (2.13–7.88%) for individual milk samples. The variability in the milk quality parameters was, therefore, also a presupposition for ensuring a large variability of the FAs and their groups. Therefore, these mentioned milk sample sets seem to be a suitable material for the validation of MFAPs by FT-MIR calibration. Thus, the validation milk sample sets represent mean proportions in terms of the MFAPs under Czech Republic dairy farm supply/demand conditions.

### 3.2. Milk FAs for Bulk and Individual Milk Samples

The highest variabilities were found for the PUFAs and TFAs in bulk milk samples, where RSDs were 46.1 and 28.2% (FT-MIR) and 19.9 and 26.4% (GC). In individual milk samples, the highest variability was found for TFA: 25.0% (FT-MIR) and 20.8% (GC) (Table 3).

Determination by FT-MIR also showed high variability for C18:1 (26.6%), while the GC assay was relatively low (9.0%) in bulk milk samples. This difference in the variability between FT-MIR and GC could be due to the fact that C18:1n-9 (*cis*-9) was determined by GC, whereas FT-MIR included a total of C18:1 isomers [43]. In individual milk samples, the differences between RSD values observed by FT-MIR and GC were not too distinct (13.3% and 17.0%, respectively). These results were probably caused by a wider range of C18:1n-9 (*cis*-9) proportion (20.7%; i.e., 12.1–32.8%) when determined by GC in comparison to bulk milk samples (8.5%; i.e., 15.2–23.7%).

Oleic acid (*cis*-9 isomer C18:1n-9) represents about 20% to 30% of milk fat and is the most abundant isomer (more than 80%) of the C18:1 group. A very heterogeneous mixture of C18:1 isomers, whether the *cis* or *trans*, is obtained by GC determination. It is also known that the variability of the proportions of *trans* isomers C18:1 is very high, ranging from 1.29 to 7.17% [47], and it depends on animal (breed, stage of lactation), feed (concentrate-to-forage ratio, fat supplementation), or management and season factors [23]. The dominant *trans*-isomer is C18:1 *trans*-11, i.e., vaccenic acid. This acid represents 24.5% to 55.1% of the total *trans*-C18:1 proportion [48]. C14:1, C16: 1, and C17:1 *trans* isomers and PUFA isomers are also present in the milk fat. In terms of PUFA isomers, C18:2 *cis*-9, *trans*-11 (conjugated linoleic acid (CLA); also known as rumenic acid) is undoubtedly the most important isomer [1,23].

In the case of GC, only the following *trans*-isomers (TFA group)—C18:1*t* (a mixture of multiple-position *trans* isomers), C18:1 *trans*-11, and C18:2 *cis*-9, *trans*-11—were identified. For bulk milk samples, the proportions of TFA were 2.5 (FT-MIR) and 2.2% (GC), respectively. For individual milk samples, TFA proportions were 2.7 (FT-MIR) and 2.3% (GC), respectively. The mean TFA values correspond to literary sources (see above), and their relatively low value was mainly due to the fact that none of the observed animals were pastured, and the cows were not fed with fresh forage or fat supplements [1,49,50].

The observation of two significant groups of FAs (SFAs vs. UFAs) showed that the variability is completely consistent with the literature [16,32,51]. Higher variability was in the UFAs than in the SFAs, both for bulk and individual milk samples. For bulk milk samples, RSDs were found for SFAs and UFAs of 5.8 and 9.2% (FT-MIR) and 4.0 and 9.6% (GC). For individual milk samples, RSDs were 5.2 and 13.9% (FT-MIR) and 5.7 and 13.8% (GC). The mean SFA and UFA values for bulk milk samples were 70.6 and 28.4% (FT-MIR) and 67.1 and 29.5% (GC). For individual milk samples, mean values were 69.5 and 29.7% (FT-MIR) and 68.1 and 28.7% (GC), respectively. These values show a high match between the two analytical methods for the determination of FAs and between bulk and individual milk samples.

The results for these two groups correspond to literature data [50,52], although the SFA values are rather high at the upper limit of the reported ranges. This fact could have been caused (as described above in the case of TFAs) by the fact that only the total mixed ration with preserved forage was used in the farms where the milk samples (bulk and individual) were collected, and pasture or fresh forage for dairy cows was not used. This is demonstrated by a high proportion of palmitic acid—C16:0: 33.7% (28.5–45.6%) for bulk milk samples (GC) and 32.2% (24.6–43.2%) for individual milk samples (GC)—as well as a low proportion of oleic acid—C18:1n-9 (*cis*-9): 19.5% (15.2–23.7%) in bulk milk samples (GC), and 19.2% (12.1–32.8%) for individual milk samples (GC). These two FAs have the highest proportion of milk fat [23,24].

### 3.3. Correlation and Regression Analysis (Assessment of FT-MIR and GC Method)

#### 3.3.1. Bulk Milk Samples

**Figure 1:**
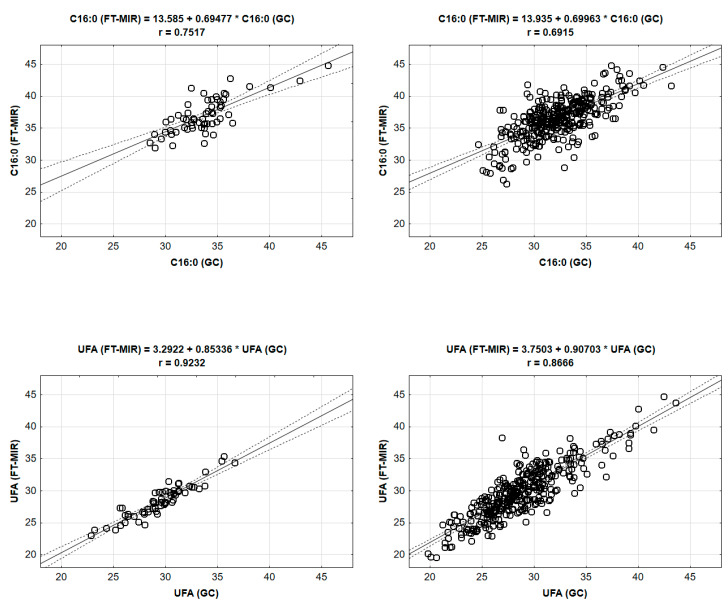
Regression analysis for palmitic acid (C16:0) and unsaturated fatty acids (UFAs) determined by gas chromatography (GC; as reference method) and mid-infrared spectroscopy (FT-MIR; as routine method) for bulk (*n* = 60; left column) and individual (*n* = 345; right column) milk samples of Holstein and Czech Fleckvieh cows—examples with data distribution.

For most FAs and their groups, very close dependencies were found, mostly at the probability level *p* < 0.001 (Table 4, Figure 1). In almost all cases, the contents of FAs determined by the FT-MIR were overestimated.

The highest differences (expressed as percentages) between the two methods were in the case of PUFAs (+47%). Regarding mean values [1,23,24,48], the more accurate PUFA proportions were determined by GC (3.5%) than those found by FT-MIR (6.6%). Nevertheless, correlation coefficients (r) were high and statistically significant (0.6278, *p* < 0.001), confirming the possibility of using the FT-MIR method. However, we have to take into account the recalculations given by the regression equations (see Tables S1 and S2). Statistically significant (*p* < 0.001) correlation coefficients were also found for C18:1 (0.4993), MUFA (0.5943), and TFA (0.5706). Additionally, the MUFA value determined by FT-MIR was overestimated (30.2%).

High consistency was found for palmitic acid (0.7517; *p* < 0.001), SFAs (0.7169; *p* < 0.001), and the UFA group (0.9232; *p* < 0.001). Less tight dependencies were found for FA groups sorted by carbon number: SCFAs (0.3308; *p* < 0.01) and MCFAs (0.3727; *p* < 0.01). In the LCFA group, the correlation was slightly higher (0.4935; *p* < 0.001).

#### 3.3.2. Individual Milk Samples

In most cases, the correlation coefficients between FT-MIR and GC for individual milk samples were higher than for bulk milk samples (Table 4, Figure 1). This fact can be due to the combination of the statistical principle and the nature of the wider variation range and thus higher variability of values of FAs in individual milk samples. The PUFA group was very similar to the bulk milk samples, i.e., the proportion determined by FT-MIR (8.1%) was +58.0% higher than that found using GC (3.4%).

Similar data (similar to bulk milk samples) were also found in the SFA and UFA groups. Both of these groups could be determined using FT-MIR with a higher degree of accuracy. This means that high consistency was found for SFAs (0.8592; *p* < 0.001) and UFAs (0.8666; *p* < 0.001). The details of the regression results are given in Tables S1 and S2.

As in the case of bulk milk samples, even in individual milk samples, low correlations were found for groups of FAs sorted by the number of carbons, particularly in the MCFA group (0.2277; *p* < 0.001). A difference (compared to bulk milk samples) was found in the LCFA group, where the correlation coefficient was surprisingly high (0.8494; *p* < 0.001). The reason for this observation is difficult to explain. In our opinion, it could be probably due to the different relative differences in bulk and individual milk samples found for MUFAs (+13.9 and +22.3%), PUFAs (+47.0 and +58.0%), and TFAs (+12.0 and 14.8%) groups, which form the basis of the LCFA group.

As compared to literature results, there are high correlation coefficients between routine and reference methods in individual or bulk milk samples [6,16,17,28,32,33]. The similar ratio (higher r in FT-MIR than in the GC method) is valid when comparing the FT-MIR and FT-NIR methods [6]. The determination of FAs by the MIR method, according to carbon chain length, is less tight (0.78) than according to saturation of FAs (0.90) [17], which is also confirmed by the values found in this work.

## 4. Conclusions

In conclusion, our results show that the FT-MIR method can be advantageously used for routine determination, mainly for those FA groups and single FAs that have a high proportion in milk fat (SFAs, UFAs, and C16:0, respectively). Taking this into consideration, this applies not only to bulk milk, but also to individual milk samples where there is a good opportunity to control or select raw milk with a nutritionally desirable milk FA composition for specific purposes.

## Figures and Tables

**Table 1:** Chromatography determination characteristics.

Characteristics	Value
Temperature: oven	55 °C—5 min, 40 °C/min—170 °C, 2 °C/min—196 °C, 10 °C/min—210 °C—8 min
Temperature: injector	250 °C
Temperature: detector	250 °C
Helium flow	1.8 mL/min
Injection	1 µL, split 10

**Table 2:** Basic statistical characteristics for selected milk quality parameters of bulk and individual milk samples of Holstein and Czech Fleckvieh cows.

Milk Parameters ^1^	Bulk Samples (n = 60)					Individual Samples (n = 345)				
	Mean	Min	Max	SD	RSD	Mean	Min	Max	SD	RSD
F	3.90	2.57	5.15	0.44	11.3	4.34	2.13	7.88	0.92	21.2
P	3.34	3.01	3.62	0.15	4.6	3.44	2.16	4.92	0.39	11.4
L	4.95	4.74	5.03	0.06	1.3	5.04	3.42	5.52	0.26	5.2
SNF	8.92	8.49	9.24	0.18	2.0	9.10	5.79	10.48	0.48	5.3
U	27.09	18.80	37.60	4.27	15.8	23.96	10.20	85.30	6.82	28.5
CA	0.180	0.164	0.200	0.009	4.9	0.190	0.117	0.261	0.023	12.3
BHB	0.027	0.010	0.050	0.016	58.0	0.030	0.010	0.500	0.039	128.5 *
AC	0.056	0.010	0.130	0.030	59.4	0.095	0.010	0.610	0.072	75.3
SCC	246	88	531	108	43.8	182	6	6463	454	248.9

SD = standard deviation; RSD = relative standard deviation in %: (SD/mean) × 100; * n = 313 for BHB. ^1^ F = fat, P = crude protein, L = lactose monohydrate, SNF = solids-non-fat (g/100 g), U = urea (mg/100 mL), CA = citric acid (%), BHB = beta-hydroxybutyric acid, AC = acetone (mmol/L), SCC = somatic cell count (thousands in mL).

**Table 3:** Basic statistical characteristics for selected fatty acids (FAs) and their groups, determined by gas chromatography (GC) and mid-infrared spectroscopy (FT-MIR), including conversion to identical GC groups, of bulk and individual milk samples of Holstein and Czech Fleckvieh cows.

FAs and Their Groups ^1^	Bulk Samples (*n* = 60)					Individual Samples (*n* = 345)				
	Mean	Min	Max	SD	RSD	Mean	Min	Max	SD	RSD
FT-MIR (g/100 g total FAs) ^2^										
C16:0	37.0	32.0	44.8	2.8	7.5	36.5	26.3	44.9	3.1	8.5
C18:0	12.8	8.4	17.8	1.6	12.7	13.4	7.9	19.7	1.8	13.4
C18:1	24.8	11.4	35.0	6.6	26.6	28.6	18.9	42.3	3.8	13.3
SFA	70.6	62.2	79.1	4.1	5.8	69.5	56.2	79.8	3.6	5.2
UFA	28.4	23.0	35.4	2.6	9.2	29.7	19.6	44.8	4.1	13.9
MUFA	30.2	20.4	40.1	5.2	17.4	32.7	22.8	45.1	3.7	11.2
PUFA	6.6	2.2	10.7	3.0	46.1	8.1	5.2	12.1	1.2	15.1
TFA	2.5	0.7	3.6	0.7	28.2	2.7	0.3	4.7	0.7	25.0
SCFA	10.0	7.1	12.8	1.3	12.8	10.1	5.7	13.5	1.4	14.1
MCFA	43.6	36.4	56.4	5.6	12.8	43.5	19.0	91.9	7.3	16.7
LCFA	34.6	23.9	53.9	5.7	16.5	35.7	26.0	55.5	5.1	14.4
GC (g/100 g total FAs)										
C16:0	33.7	28.5	45.6	3.0	8.9	32.2	24.6	43.2	3.1	9.6
C18:0	8.9	5.1	11.8	1.1	12.0	9.3	4.5	17.5	1.9	20.6
C18:1n-9 (*cis*-9)	19.5	15.2	23.7	1.7	9.0	19.2	12.1	32.8	3.3	17.0
SFA	67.1	60.3	73.8	2.7	4.0	68.1	54.1	77.2	3.8	5.7
UFA	29.5	22.8	36.7	2.8	9.6	28.7	19.8	43.6	3.9	13.8
MUFA	26.0	20.6	31.3	2.3	8.8	25.4	17.2	40.3	3.7	14.6
PUFA	3.5	2.0	5.4	0.7	19.9	3.4	2.1	4.7	0.5	14.4
TFA	2.2	1.0	4.5	0.6	26.4	2.3	1.3	4.3	0.5	20.8
SCFA	11.6	9.6	13.8	1.0	8.3	13.2	5.3	19.9	2.1	16.2
MCFA	52.0	45.0	65.0	3.5	6.6	50.8	36.9	62.1	4.2	8.3
LCFA	36.4	24.6	44.7	3.5	9.5	36.0	25.1	57.6	5.3	14.6

SD = standard deviation; RSD = relative standard deviation in %: (SD/mean) × 100. ^1^ SFA = saturated FAs, UFA = unsaturated FAs, MUFA = monounsaturated FAs, PUFA = polyunsaturated FAs, TFA = *trans* isomers of unsaturated FAs, SCFA = short-chain FAs, MCFA = medium-chain FAs, LCFA = long-chain FAs. ^2^ FT-MIR = FAs and their groups calculated as FAs determined by the FT-MIR (g/100 g in milk) × 100/fat determined by the FT-MIR × 0.95.

**Table 4:** Comparison of fatty acids (FAs) and their groups determined by gas chromatography (GC; as reference method) and mid-infrared spectroscopy (FT-MIR; as routine method) of bulk and individual milk samples of Holstein and Czech Fleckvieh cows.

FAs and Their Groups ^4^	Bulk Samples (*n* = 60)							Individual Samples (*n* = 345)						
	g/100 g Total FAs				Correlation Analysis ^1^			g/100 g Total FAs				Correlation Analysis ^1^		
	FT-MIR ^2^	GC	Difference ^3^		*p* (*t*-test)	r	*p*	FT-MIR ^2^	GC	Difference ^3^		*p* (*t*-test)	r	*p*
			abs.	rel.						abs.	rel.			
C16:0	37.0	33.7	+3.3	+8.9	<0.001	0.7517	<0.001	36.5	32.2	+4.3	+11.8	<0.001	0.6915	<0.001
C18:0	12.8	8.9	+3.9	+30.5	<0.001	0.5459	<0.001	13.4	9.3	+4.1	+30.6	<0.001	0.6718	<0.001
C18:1 ^5^	24.8	19.5	+5.3	+21.4	<0.001	0.4993	<0.001	28.6	19.2	+9.4	+32.9	<0.001	0.7813	<0.001
SFA	70.6	67.1	+3.5	+5.0	<0.001	0.7169	<0.001	69.5	68.1	+1.4	+2.0	<0.001	0.8592	<0.001
UFA	28.4	29.5	−1.1	−3.9	0.0413	0.9232	<0.001	27.8	28.7	−0.9	−3.2	<0.001	0.8666	<0.001
MUFA	30.2	26.0	+4.2	+13.9	<0.001	0.5943	<0.001	32.7	25.4	+7.3	+22.3	<0.001	0.7580	<0.001
PUFA	6.6	3.5	+3.1	+47.0	<0.001	0.6278	<0.001	8.1	3.4	+4.7	+58.0	<0.001	0.3314	<0.001
TFA	2.5	2.2	+0.3	+12.0	0.0531	0.5706	<0.001	2.7	2.3	+0.4	+14.8	<0.001	0.1690	<0.01
SCFA	10	11.6	−1.6	−16.0	<0.001	0.3308	<0.01	10.1	13.2	−3.1	−30.7	<0.001	0.5645	<0.001
MCFA	43.6	52.0	−8.4	−19.3	<0.001	0.3727	<0.01	43.5	50.8	−7.3	−16.8	<0.001	0.2277	<0.001
LCFA	34.6	36.4	−1.8	−5.2	0.0450	0.4935	<0.001	35.7	36.0	−0.3	−0.8	<0.001	0.8494	<0.001

^1^ r = correlation coefficient, *p* = (significant level). ^2^ FT-MIR = FAs and their groups calculated as FAs determined by the FT-MIR (g/100 g in milk) × 100/fat determined by the FT-MIR × 0.95. ^3^ abs. = absolute difference (g/100 g all FAs), rel. = relative difference calculated as 100—((GC × 100)/FT-MIR). ^4^ SFA = saturated FAs, UFA = unsaturated FAs, MUFA = monounsaturated FAs, PUFA = polyunsaturated FAs, TFA = *trans* isomers of unsaturated FAs, SCFA = short-chain FAs, MCFA = medium-chain FAs, LCFA = long-chain FAs. ^5^ for GC C18:1n-9 (*cis*-9).

## Supplementary Materials

The following are available online at https://www.mdpi.com/2076-2615/10/6/1095/s1, Table S1: Comparison of milk fatty acids (FAs) and their groups determined by gas chromatography (GC; as reference method) and mid-infrared spectroscopy (FT-MIR; as routine method); results of regression analysis: regression equation (Y = A + B × X1; where A = intercept, B = slope coefficient, SE = standard error; X1 = particular FAs determined by GC), Table S2: Comparison of milk fatty acids (FAs) and their groups determined by gas chromatography (GC; as reference method) and mid-infrared spectroscopy (FT-MIR; as routine method); results of regression analysis: regression equation (Y = B × X1; where B = slope coefficient, SE = standard error; X1 = particular FAs determined by GC).
