# Supplementary material for: Comparison of Fatty Acid Proportions Determined by Mid-Infrared Spectroscopy and Gas Chromatography in Bulk and Individual Milk Samples
Source: Animals (Basel). 2020 Jun 25;10(6):1095. doi: 10.3390/ani10061095 (PMC7341201; doi:10.3390/ani10061095)
Supplement: Supplementary file 1 [file animals-10-01095-s001.pdf]

Table S1. Comparison of milk fatty acids (FAs) and their groups determined by gas chromatography (GC; as reference method) and mid-infrared spectroscopy (FT-MIR; as routine method); results of regression analysis: regression equation (**$Y = A + B \times X_1$**; where A = intercept, B = slope coefficient, SE = standard error; X_1 = particular FAs determined by GC), coefficient of determination (R^2), rank correlation (SC = Spearman coefficient), standard deviation of predicted values (SD pred.), difference between standard deviation of observed and predicted values (SD diff.).

**FAs [1] and their groups**	**Bulk samples (n = 60)**											**Individual samples (n = 345)**										
	A	SE (A)	*P*-value (A)	B	SE (B)	*P*-value (B)	R^2	SC	*P*-value (SC)	SD pred.	SD diff.	A	SE (A)	*P*-value (A)	B	SE (B)	*P*-value (B)	R^2	SC	*P* -value (SC)	SD pred.	SD diff.
C16:0	13.6	2.71	<0.001	0.69	0.08	<0.001	0.57	0.67	<0.001	2.08	0.69	13.9	1.28	<0.001	0.70	0.04	<0.001	0.48	0.64	<0.001	2.15	0.96
C18:0	5.4	1.50	<0.001	0.83	0.17	<0.001	0.30	0.35	<0.01	1.62	0.80	7.5	0.36	<0.001	0.63	0.04	<0.001	0.45	0.60	<0.001	1.21	0.59
C18:1 [2]	-12.0	8.41	0.159	1.89	0.43	<0.001	0.25	0.63	<0.001	3.29	3.30	11.2	0.76	<0.001	0.91	0.04	<0.001	0.61	0.72	<0.001	2.96	0.83
SFA	-2.9	9.39	0.759	1.09	0.14	<0.001	0.51	0.70	<0.001	2.93	1.16	14.8	1.76	<0.001	0.80	0.03	<0.001	0.74	0.84	<0.001	3.10	0.51
UFA	3.3	1.38	0.020	0.85	0.05	<0.001	0.85	0.91	<0.001	2.42	0.20	3.8	0.82	<0.001	0.91	0.03	<0.001	0.75	0.84	<0.001	3.58	0.55
MUFA	-5.4	6.35	0.398	1.37	0.24	<0.001	0.35	0.67	<0.001	3.12	2.13	13.7	0.89	<0.001	0.75	0.03	<0.001	0.57	0.72	<0.001	2.78	0.89
PUFA	-3.0	1.59	0.065	2.77	0.45	<0.001	0.39	0.72	<0.001	1.91	1.13	5.3	0.44	<0.001	0.84	0.13	<0.001	0.11	0.35	<0.001	0.41	0.82
TFA	1.0	0.29	0.002	0.67	0.13	<0.001	0.33	0.63	<0.001	0.40	0.30	2.2	0.18	<0.001	0.24	0.08	0.002	0.03	0.25	<0.001	0.11	0.56
SCFA	4.9	1.91	0.013	0.44	0.16	<0.001	0.11	0.28	<0.05	0.42	0.85	5.2	0.40	<0.001	0.37	0.03	<0.001	0.32	0.57	<0.001	0.80	0.62
MCFA	12.4	10.23	0.232	0.60	0.20	0.003	0.14	0.51	<0.001	2.07	3.49	23.5	4.62	<0.001	0.39	0.09	<0.001	0.05	0.32	<0.001	1.66	5.62
LCFA	5.0	6.87	0.467	0.81	0.19	<0.001	0.24	0.59	<0.001	2.82	2.90	6.1	1.02	<0.001	0.82	0.03	<0.001	0.71	0.78	<0.001	4.33	0.80

[1] SFA = saturated FAs, UFA = unsaturated FAs, MUFA = monounsaturated FAs, PUFA = polyunsaturated FAs, TFA = *trans* isomers of unsaturated FAs, SCFA = short-chain FAs, MCFA = medium-chain FAs, LCFA = long-chain FAs.

[2] for GC C18:1n-9 (*cis*-9).

Table S2. Comparison of milk fatty acids (FAs) and their groups determined by gas chromatography (GC; as reference method) and mid-infrared spectroscopy (FT-MIR; as routine method); results of regression analysis: regression equation (**$Y = B \times X_1$**; where B = slope coefficient, SE = standard error; X_1 = particular FAs determined by GC), coefficient of determination (R^2), rank correlation (SC = Spearman coefficient), standard deviation of predicted values (SD pred.), difference between standard deviation of observed and predicted values (SD diff.).

**FAs [1] and their groups**	**Bulk samples (n = 60)**										
	A	SE (A)	*P*-value (A)	B	SE (B)	*P*-value (B)	R^2	SC	*P*-value (SC)	SD pred.	SD diff.
C18:1[2]	-	-	ns	1.28	0.04	<0.001	0.949	0.63	<0.001	2.23	4.37
SFA	-	-	ns	1.05	0.01	<0.001	0.998	0.70	<0.001	2.81	1.27
MUFA	-	-	ns	1.16	0.02	<0.001	0.981	0.67	<0.001	2.65	2.60
PUFA	-	-	ns	1.94	0.09	<0.001	0.889	0.72	<0.001	1.33	1.70
MCFA	-	-	ns	0.84	0.01	<0.001	0.986	0.51	<0.001	2.89	2.67
LCFA	-	-	ns	0.95	0.02	<0.001	0.980	0.59	<0.001	3.30	2.42

[1] SFA = saturated FAs, MUFA = monounsaturated FAs, PUFA = polyunsaturated FAs, MCFA = medium-chain FAs, LCFA = long-chain FAs.

[2] for GC C18:1n-9 (*cis*-9).
